# Broadening the spectrum of cancer genes under selection in human populations

**DOI:** 10.1096/fba.2020-00150

**Published:** 2021-03-27

**Authors:** Konstantinos Voskarides

**Affiliations:** ^1^ Medical School University of Cyprus Nicosia Cyprus

There is an increasing accumulation of evidence showing that the frequency of many human diseases has been formed by evolutionary procedures. Siddiqui et al.^1^ performed an amazing study published in this journal, showing that *SIGLEC12* gene expression is associated with advanced cancer progression in humans. They performed a multi‐level experimental and statistical analysis. In a multi‐tissue carcinoma array, the gene product was present in ~80% of tissues and only 35% in a set of normal epithelial tissues. Forced expression of the gene in a *SIGLEC12* null carcinoma cell line had the consequence of enriching the transcription of genes associated with cancer progression, especially those related with KRAS and YAP/TAZ pathways. Compared with chimpanzees, it seems that the human receptor Siglec12 has lost its ligand after the human/chimpanzee lineage split, but notably the ligand gene remained in the chimpanzee genome. The authors also found evidence, using the Fst and Tajima D statistics, that a *SIGLEC12* null allele in human populations, ranging from 38% in sub‐Saharan Africans to 86% in native American populations, is under positive selection. They propose that the functional *SIGLEC12* gene is currently under an elimination process in human populations, since its encoded protein, for unknown reasons, activates carcinogenic pathways.

This study of Siddiqui et al.^1^adds to previous studies that showed significant evidence for the action of natural selection on cancer genes. All human populations have high incidences of cancer, but some have especially much higher risk for certain cancer types. Voskarides^2^ has presented data showing that populations living in extreme cold environments and extreme high altitudes have the highest cancer incidence in the world. Linear regression analysis supports the fact that the cancer‐cold association is a world‐wide phenomenon. Analysis of 247 Genome Wide Association Studies (GWAS) showed that this association has a genetic base. Cancer genes and especially tumor suppressor genes were found to be under selection in Inuit, Eskimos and Alaska Indians. Genetic variants that contributed to the survival of humans in extreme environments are probably associated with cancer incidence today. A similar evolutionary process was revealed for human populations living at extreme high altitudes, like Ethiopians and Andeans (high cancer rates, tumor suppressor genes under selection). A supporting fact is that previous studies showed that p53 mutations causing cancer in humans were found to be significant for the survival of some mammal species in the Tibetan highlands. These results were published in 2004 and 2013 by two different research teams. They found that in three different rodent species living under extreme environmental conditions (cold, hypoxia), certain amino acids in two conserved p53 residue positions (R174, S106) render p53 unable to activate apoptosis pathways.^3^, ^4^ It seems that resistance to apoptosis, a carcinogenic trait, it's an advantage under certain environmental conditions. This is related to a special evolutionary process known as "antagonistic pleiotropy", first proposed by George Williams,^5^ one of the most well‐known evolutionary biologists of the past century. He suggested that genetic variants selected as beneficial for survival at young ages, may have a deleterious effect later in life.^6^, ^7^ Selection of deleterious variants in DNA repair genes (considered as one of the main classes of tumor suppressor genes in mammals) is vital for unicellular organisms escaping extinction. Bacteria, protozoa, and monocellular fungi become very resistant under stressful conditions (antibiotics, oxidative stress, nutrient limitation, and other) when carrying mutations in DNA repair genes like *MSH2*, *PMS1*, *RAD51*, and others.^8^, ^9^, ^10^, ^11^ When these genes malfunction, the mutation flow is increasing and so are the probabilities for a beneficial mutation to appear. Weak selection on certain traits (the trait here is mutagenesis rate) is called relaxed selection. When environmental conditions come back to normal, these mutations can be gradually decreased or eliminated from unicellular populations.^12^

Has this phenomenon remained conserved in multicellular organisms? Martincorena et al.^13^ has found thousands of deleterious mutations in cells of healthy esophageal tissue from healthy individuals, in 14 well‐known cancer genes. Further analysis showed that these genes are under strong positive selection. The two most mutated genes are *NOTCH1* and *TP53*, genes that are frequently found mutated in esophageal cancer. Similar findings were published afterwards for other human tissues.^14^, ^15^, ^16^ Maybe we have to re‐consider our beliefs about deleteriousness of mutations in cancer genes. Our cells, especially those living under challenging conditions, probably accumulate deleterious mutations in cell cycle, apoptosis and DNA repair genes, in order to resist toxic agents and survive. There are also examples of genetic variants that appear to involve cancer risk and reproduction. Every biologist knows that mutations in the *BRCA1*/*2* genes can cause ovarian and breast cancer. Utah women carrying pathogenic mutations in *BRCA1*/*2* genes and living under natural fertility conditions (born before 1930) had more children, shorter interbirth intervals, later age at last birth, and higher post‐reproductive mortality than healthy controls.^17^ This pattern was absent in women living in environments with modern contraception. Scientists trying to confirm these data, found mixed results.^18^, ^19^, ^20^ Evidence of positive selection has been found only for the *BRCA2* gene.^21^

Summarizing, I think that it is presently a fact that cancer is an 100% evolutionary phenomenon. Modern studies allow scientists to realize the action of natural selection on cancer genes, cancer cells, individuals, and populations. Unfortunately, it is very hard for natural selection to eliminate mutations that cause cancer after the end of the reproductive age. It is even harder when cancer mutations have a dual effect by increasing adaptation. Taking examples from unicellular organisms,^12^ it is possible that these deleterious mutations appear and disappear through a cycle procedure, that we poorly understand and is not yet proven in multicellular organisms. If it occurs, this cycle is spread over hundreds of thousands of years, thus it is difficult to study and confirm. Extreme events like famine, pandemics, radical climate change etc. can disrupt this cycle and mutation‐selection equilibria may take too long to be stabilized. Maybe we have to find suitable animal models to simulate evolution in the laboratory to understand further these procedures.

## References

[1] Siddiqui SS , Vaill M , Do R , et al. Human‐specific polymorphic pseudogenization of SIGLEC12 protects against advanced cancer progression. FASEB BioAdvances. 2021;3(2):69–82. 33615152 10.1096/fba.2020-00092 PMC7876704

[2] Voskarides K . Combination of 247 genome‐wide association studies reveals high cancer risk as a result of evolutionary adaptation. Mol Biol Evol. 2018;35(2):473‐485. 10.1093/molbev/msx305 29220501 PMC5850495

[3] Zhao Y , Ren J‐L , Wang M‐Y , et al. Codon 104 variation of p53 gene provides adaptive apoptotic responses to extreme environments in mammals of the Tibet plateau. Proc Natl Acad Sci. 2013;110(51):20639‐20644. 10.1073/pnas.1320369110 24297887 PMC3870672

[4] Ashur‐Fabian O , Avivi A , Trakhtenbrot L , et al. Evolution of p53 in hypoxia‐stressed Spalax mimics human tumor mutation. Proc Natl Acad Sci. 2004;101(33):12236‐12241. 10.1073/pnas.0404998101 15302922 PMC514462

[5] Rodríguez JA , Marigorta UM , Hughes DA , Spataro N , Bosch E , Navarro A . Antagonistic pleiotropy and mutation accumulation influence human senescence and disease. Nat Ecol Evol. 2017;1(3): 10.1038/s41559-016-0055 28812720

[6] Austad SN , Hoffman JM . Is Antagonistic Pleiotropy Ubiquitous in Aging Biology? Evol Med Public Heal. 2018;2018(1):287‐294. 10.1093/emph/eoy033 PMC6276058 30524730

[7] Byars SG , Voskarides K . Genes that improved fitness also cost modern humans: Evidence for genes with antagonistic effects on longevity and disease. Evol Med Public Heal. 2019;2019(1): 10.1093/emph/eoz002 PMC6379702 30799869

[8] LeClerc JE , Li B , Payne WL , Cebula TA . High mutation frequencies among Escherichia coli and Salmonella pathogens. Science (80‐). 1996;274(5290):1208‐1211. 10.1126/science.274.5290.1208 8895473

[9] Grazielle‐Silva V , Zeb TF , Bolderson J , et al. Distinct phenotypes caused by mutation of MSH2 in trypanosome insect and mammalian life cycle forms are associated with parasite adaptation to oxidative stress. PLoS Negl Trop Dis. 2015;9(6):e0003870. 10.1371/journal.pntd.0003870 26083967 PMC4470938

[10] Healey KR , Zhao Y , Perez WB , et al. Prevalent mutator genotype identified in fungal pathogen Candida glabrata promotes multi‐drug resistance. Nat Commun. 2016;7: 10.1038/ncomms11128 PMC5603725 27020939

[11] Byrne RT , Klingele AJ , Cabot EL , et al. Evolution of extreme resistance to ionizing radiation via genetic adaptation of DNA repair. Elife. 2014;3: 10.7554/elife.01322 PMC3939492 24596148

[12] Giraud A , Radman M , Matic I , Taddei F . The rise and fall of mutator bacteria. Curr Opin Microbiol. 2001;4(5):582‐585. 10.1016/S1369-5274(00)00254-X 11587936

[13] Martincorena I , Fowler JC , Wabik A , et al. Somatic mutant clones colonize the human esophagus with age. Science (80‐). 2018;362(6417):911‐917. 10.1126/science.aau3879 PMC6298579 30337457

[14] Lee‐Six H , Øbro NF , Shepherd MS , et al. Population dynamics of normal human blood inferred from somatic mutations. Nature. 2018;561(7724):473‐478. 10.1038/s41586-018-0497-0 30185910 PMC6163040

[15] Salk JJ , Loubet‐Senear K , Maritschnegg E , et al. Ultra‐sensitive TP53 sequencing for cancer detection reveals progressive clonal selection in normal tissue over a century of human lifespan. Cell Rep. 2019;28(1):132‐144.e3. 10.1016/j.celrep.2019.05.109 31269435 PMC6639023

[16] Yizhak K , Aguet F , Kim J , et al. RNA sequence analysis reveals macroscopic somatic clonal expansion across normal tissues. Science (80‐). 2019;364(6444):eaaw0726‐ 10.1126/science.aaw0726 PMC7350423 31171663

[17] Smith KR , Hanson HA , Mineau GP , Buys SS . Effects of BRCA1 and BRCA2 mutations on female fertility. Proc R Soc B Biol Sci. 2012;279(1732):1389‐1395. 10.1098/rspb.2011.1697 PMC3282366 21993507

[18] Friedman E , Kotsopoulos J , Lubinski J , et al. Spontaneous and therapeutic abortions and the risk of breast cancer among BRCA mutation carriers. Breast Cancer Res. 2006;8(2): 10.1186/bcr1387 PMC1557713 16563180

[19] Moslehi R , Singh R , Lessner L , Friedman JM . Impact of BRCA mutations on female fertility and offspring sex ratio. Am J Hum Biol. 2010;22(2):201‐205. 10.1002/ajhb.20978 19642207 PMC3739697

[20] Pal T , Keefe D , Sun P , Narod SA . Fertility in women with BRCA mutations: a case‐control study. Fertil Steril. 2010;93(6):1805‐1808. 10.1016/j.fertnstert.2008.12.052 19200971

[21] Vicens A , Posada D . Selective pressures on human cancer genes along the evolution of mammals. Genes (Basel). 2018;9(12): 10.3390/genes9120582 PMC6316132 30487452

